# Using community participation to assess demand and uptake of scaling and polishing in rural and urban environments

**DOI:** 10.1186/s12903-018-0548-9

**Published:** 2018-05-10

**Authors:** Ezi A. Akaji, Nkolika P. Uguru, Sam N. Maduakor, Etisiobi M. Ndiokwelu

**Affiliations:** Department of Preventive Dentistry, Faculty of Dentistry, College of Medicine, University of Nigeria, UNTH, Enugu, Nigeria

**Keywords:** Periodontal disease, Community health education, Dental scaling, Utilization, Health services demand

## Abstract

**Background:**

One of the control tools for periodontal disease besides individual home care is professional oral prophylaxis that is, Scaling and Polishing (S&P).The aim of this study is to assess the effect of oral health awareness on the demand and uptake of scaling and polishing among dwellers of rural and urban environments.

**Methods:**

This interventional study was conducted in Enugu, Nigeria. A questionnaire was used to obtain data on demographic details, presenting complaints and requests, and prior dental visits from consenting attendees in 4 community outreaches. The number of those demanding for scaling of teeth at point of presentation was extracted from their requests. Oral health talk was then given as the intervention for the study. Periodontal assessment was done using Community Periodontal index (CPI) and participants who received scaling thereafter were recorded. Data were analyzed with SPSS [version 20] employing Chi square to compare categorical variables and p was significant at ≤0.05. Multiple regression analysis of factors affecting oral health awareness was done and outcome of intervention was determined by percentage difference in number of participants demanding and receiving S&P.

**Results:**

A total of 454 participants enlisted for the study. The outreaches served as first point of contact with dental professionals for 383 (84.4%) participants. 60 (80%) and 15 (20%) participants demanded for scaling in the urban and rural locations respectively (*p* = 0.00). Out of 78 with CPI 3 score, only 8 (10.3%) demanded for S&P but uptake was by 73 (93.6%) [p = 0.00]. Outcome of oral health intervention was 80.6% difference among those with periodontitis. Multiple regression analysis of factors showed that participants’ locations, that is, rural or urban, was the only factor that significantly affected oral health awareness (C.*I* = 0.183–0.375, *p* = 0.000).

**Conclusion:**

Demand for scaling was sub-optimal but the uptake was satisfactory. Rural or urban location of the participants significantly influenced their oral health awareness. The keenness to take up scaling suggests benefits accruing from the oral health education. Appropriate health policies and planning could help bridge the gap between rural and urban areas and strengthen gains from this study.

**Electronic supplementary material:**

The online version of this article (10.1186/s12903-018-0548-9) contains supplementary material, which is available to authorized users.

## Background

The term periodontal disease encompasses all pathological conditions of periodontal tissues categorized broadly as gingivitis and periodontitis [1].Gingivitis, an inflammatory lesion of marginal gingiva is highly prevalent in most populations and at most ages with global values ranging from 50 to 90% [2]. Gingivitis could resolve with improved oral hygiene, persist indefinitely or may result in attachment loss. [3] If loss or destruction of periodontal attachment or alveolar bone occurs, the condition is characterized as periodontitis [4]. Periodontitis is a major public health problem having met all the conditions for such, and the manifestations –bleeding, halitosis, gingival recession and tooth loss impact negatively on the affected individual [5, 6]. It is the most common chronic inflammatory disease seen in humans, affecting nearly half of adults in the United Kingdom and 60% of those over 65 years [7].Severe periodontitis, which may result in tooth loss, is found in most populations affecting both old and young age groups [8, 9]. A systematic analysis of global burden of oral conditions from 1990 to 2010 by Marcenes et al. (2013) showed that, severe periodontitis was the leading cause of Disability Adjusted Life Years (DALYs) in 9 regions of the world -Australia, Sub-Saharan Africa, East, Central, East, and Southeast Asia, and Southern, Central, Tropical, and Latin America [10]. A similar study specific for periodontitis found the disease had affected 743 million people worldwide with the age-standardized incidence rate of 701 cases per 100,000 person/years for severe periodontitis in 2010 [11].

The primary aetiological factor for periodontitis is dental plaque, which is a tenaciously adherent biofilm on teeth and gingival surfaces and is 70% bacteria while mineralized plaque deposits called calculus is one of the secondary factors [1]. Professional oral prophylaxis (that is scaling and polishing) performed by a dental care professional serves as one control measure for the disease [12]. Scaling is the removal of plaque, calculus, debris and staining from the crown and root surfaces of the teeth using specially designed sharp dental hand instruments or ultrasonic scalers. In order to smoothen teeth surfaces, a procedure called polishing is carried out. This involves removing any residual extrinsic stains and deposits using a rubber cup or bristle brush loaded with a prophylactic paste [12]. Scaling and polishing is nonsurgical procedure; intended to supplement the patient’s home-care plaque control and is frequently provided as part of the dental recall appointment [13].With the removal of plaque and calculus, the clinical indicators of the active disease - bleeding and inflammation of the gums (gingivitis) – are also reduced and over time a reduction in gingivitis will reduce progression to periodontitis [12]. Individuals could benefit from this basic dental care every 6, 9 or 12 months depending on their peculiar needs [12, 13].Sadly, most do not access this basic dental care with negative implications to their periodontal health and this is often a function of level of awareness of oral diseases [14].

Access has being defined by several authors from different dimensions [15]. Utilization often used as a proxy of access, (that is, realised access) [16], is influenced by the supply as well as the demand for services, including individual attributes such as preferences, tastes and information [15].An individual or collective alertness to the existence and prevention of oral diseases and an equal alertness in taking necessary steps to obtain treatment for these diseases when they occur is referred to as oral health awareness [17]. According to some studies, poor oral health awareness among other factors is responsible for the occurrence of dental diseases [14, 18]. Other studies observed that when there is low oral health awareness, there is a direct effect on the illness seeking behaviour of the individual and population. The most common scenario is the underutilization of oral health facilities and/or late presentation at the clinic with resultant complications due to poor public enlightenment regarding prevention and treatment of these diseases [14].The concept of demand, utilization and awareness of dental services are all interrelated. Oral health utilization can be defined as the actual attendance by members of the public at oral health facilities to receive care. Demand on the other hand, ‘which equates demand for uptake in our study’ can be defined as a perception by the individual or community that a need exists. It can also be defined as the ability of the patient to seek dental health [19].

Observations from a study conducted in rural India show a significant association between dental awareness and demand for dental services [18]. Another study reported that awareness of individuals and communities need to be built in order to motivate the use of dental services. The same study also observed that imparting preventive dental education and strengthening of dental health facilities will increase utilization. It was also concluded that barriers to demand for dental care and utilization of dental services can be removed by motivating people and creating awareness about oral health problems prevention strategies and treatment in order to remove fear and anxiety [20]. Therefore, we hypothesize that if oral health education were provided to individuals to increase their oral health awareness, then, there will be increase in demand and uptake of oral health preventive services.

For a wider view on demand and uptake of scaling services, we conducted our study in rural and urban communities using outreach platforms. Community outreach programs offer opportunities for early diagnosis and treatment, dental health education, and institution of preventive measures so can spread awareness and disseminate treatment thereby enhancing access to care especially within the rural communities [21, 22].The aim of our study was to assess the effect of oral health awareness on the demand and uptake of scaling and polishing among dwellers of rural and urban environments.

## Methods

### Study area and design

This was an interventional study conducted in Enugu State Nigeria. The state is situated in inland south eastern Nigeria, and covers 7161 km^2^ (Additional file 1). It is made up of 17 local government areas (LGAs) with at least one primary health care centre sited in each LGA [23]. From the list of LGAs obtained, we grouped them according to their geographic location (urban and rural). We selected two LGAs each from urban and rural areas by simple random sampling. This was done to ensure proper representation of both urban and rural communities. From each selected LGA, a community was selected by simple random method from the list of communities provided. Outreach was staged in each selected community and the Thailand’s oral care model described by Professor P. Phantumvanit in the Peterson and Kwam’s study (2004) was partly adopted here [24].The model fosters community participation and capacity building for oral health promotion, hence, for our study, each selected community provided volunteers to help with the program.

### Sample size and selection of participants

A minimum sample size of 453 was obtained using the formula N = Z^2^ P (1-P)/D^2^ with a consideration of the possibility of 20% non-response from participants (Araoye 2003) [25]. Our sample frame was drawn from households residing in the communities where outreach programs were held. Community heads and volunteers they endorsed facilitated the process by informing and inviting households and individuals to the outreach.

### Inclusion and exclusion criteria

Consenting attendees who were willing to participate in study (see Additional file 2) were included while those who gave no consent were excluded.

### Data collection

#### Phase 1

As attendees arrived at the outreach, a questionnaire (Additional file 2) was administered to the consenting ones by the researchers to obtain data on demographic details, frequency of tooth brushing and number of visits to an oral health care professional prior to the outreach and present dental concern/need if any. One of the options for dental concern /need stem question was “cleaning of teeth”. Response to this question was used to measure demand for S&P. Thereafter, an oral health educational talk highlighting the need and methods for good oral care with tooth brushing demonstrations and the attendant implications were done. Volunteers translated the talk in the native dialect of the community. The oral health education (OHE) served as the intervention in this study. All attendees (study participants and others) were given tooth cleaning materials. Phase 1 ended with periodontal assessment of consenting participants. This was done by 3 examiners employing the modified Community Periodontal Index (CPI) [26] with a sterilized CPI probe and mouth mirror under artificial illumination provided by fluorescent light and head banded torch, in a temporarily created screened corner at the outreach venue. Face mask and examination gloves were worn by the examiner to prevent cross infection. Calibration of examiners was done by training them on the specific instruments to use. There was pre-assessment on 10 patients before the outreach in order to determine the uniformity of instruments of measure (CPI probe) and the grading system used by all examiners. The examiners were asked to measure and chart findings during the training and were assessed to ensure reliability and validity of measurement. Probing depth was used as the unit of measure among the three examiners.

#### Phase 2

Here, uptake of scaling and polishing (S&P) was recorded capturing those that requested for dental scaling when the questionnaire was administered initially and those who decided for it after the oral health intervention. The uptake was measured by the number of participants receiving S&P at the outreach or 6 months after in designated oral health care centres. S&P was carried out by dentists, dental therapists and final year dental students using disposable materials and instruments in separate packs for each participant in accordance with universal infection control standards prescribed by the World Health Organization [27]. The scaling procedure was done with the participants seated on a mobile dental chair which had a detachable sputum bowl. To ensure privacy, a collapsible standing shield was used to secure the place. Hand instruments such as jacquette scalers, and universal scalers were used to remove dental plaque and calculus from tooth surfaces; the recipients rinsed their mouth with water from disposable cups intermittently. Those who opted to have their scaling done later were given identifiers in form of tally numbers and sent to designated dental centres.

Our primary outcome was determined by percentage difference in demand and uptake of scaling services amongst those with scaling treatment need after delivery of the oral health education. For the purpose of this study, awareness was measured using number of dental visits as proxy for it. We did that because evidence from studies carried out in similar context show that the number of dental visits made by respondents for routine check up or otherwise was based on their level of awareness [14, 20, 28].

### Data analysis

Data collected were analysed using the Statistical Package for Social Sciences software for windows (version 20 SPSS Inc. Chicago IL), describing categorical variables using frequencies and percentages. Statistical significant differences among groups were determined by the Chi-square test and Confidence intervals; the level of significance was set at *P* ≤ 0.05. Percentage difference in demand and uptake of scaling of teeth was calculated.

## Results

Four hundred and fifty four participants aged 2 to 86 years (mean: 38.5 ± 14.8 years) were included in the study. 245 (54.0%) participants enlisted in the rural outreaches and 209 (46.0%) in the urban centres; 219 (48.2%) were males and 235 (51.8%) were females. The outreaches served as first point of contact with dental professionals for 383 (84.4%) participants and as second for 56 (12.3%). Other demographic details are in Table 1.

**Table 1:** Socio-demographic details of the participants

Participants’ characteristics	Number (*N* = 454)	Percentage
Age group		
0–29 yrs	152	33.5%
30–59 yrs	255	56.2%
≥ 60 yrs. (Mean: 38.5 ± 14.8 yrs)	47	10.3%
Gender		
Male	219	48.2
Female	235	51.8
Location		
Urban	209	46.0
Rural	245	54.0
No of previous visit(s) for dental care		
1st visit	383	84.4
2nd visit	56	12.3
> 2 visits	15	3.3
Demand for dental scaling		
Yes	75	16.5
No	379	83.5
Uptake of dental scaling		
Yes	364	80.2
No	90	19.8

The outreach served as first contact with dental professionals for 84.4% of the participants

On the overall, 60 (28.7%) participants demanded for scaling of their teeth in the urban outreaches and 15(6.1%) did in the rural communities (*p* = 0.00). Also, 50 (66.7%) males and 25 (33.7%) females demanded for scaling at the outreaches (p = 0.00). A total of 364(80.2%) participants had scaling and polishing during the program and/or within 6 months after in designated dental facilities; 160 (44.0%) in the urban and 204 (56.0%) in the rural communities (*p* = 0.04).These are shown in Table 2.

**Table 2:** Level of demand and uptake of dental scaling according to location and gender

Variable	Demand		Uptake	
	Yes	No	Yes	No
Urban	60 (80%)	149 (39.3%)	160 (44%)	49 (54.4%)
Rural	15 (20%)	230 (60.7%)	204 (56%)	41 (45.6%)
Total	75 (100%)	379 (100)	364 (100)	90 (100)
*p*-value	*p* = 0.00*		*p* = 0.07	
Male	50 (66.7%)	169 (44.6%)	175 (48.1%)	44 (48.9%)
Female	25 (33.3%)	210 (55.4%)	189 (51.9%)	46 (51.1%)
Total	75 (100)	379 (100)	364 (100)	90 (100)
	*p* = 0.00*		*p* = 0.89	

*Statistically significant

We also considered the association between periodontal status with demand and uptake of scaling as presented in Table 3 and found 42 (26.8%) of those with mild gingivitis [CPI score1] demanded for S&P. However, even with CPI scores 3 and 4, 70 (89.7%) and 15 (93.8%) respectively did not indicate interest in S&P (*p* = 0.00). However, 73 (93.6%) of those with CPI 3 received scaling post intervention. From the Table 3, we extracted data for percentage difference in demand and uptake of scaling amongst those with the periodontal treatment needs and obtained an 80.8% difference; details of the calculation are in Table 4.

**Table 3:** Demand and uptake of dental scaling according to periodontal status

Variable	Periodontal status of participants					
Demand for S&P	Healthy Periodontium	Mild gingivitis	Moderate Gingivitis	Periodontitis		Total
	CPI 0	CPI 1	CPI 2	CPI 3	CPI 4	
	*n* (%)	*n* (%)	*n* (%)	*n* (%)	*n* (%)	*N*
Yes	1 (7.7))	42 (26.8)	23(12.1)	8 (10.3)	1 (6.3)	75
No	12 (92.3)	115 (73.2)	167 (87.9)	70 (89.7)	15 (93.8)	379
Total	13 (100)	157 (100)	190 (100)	78 (100)	16 (100)	454
X^2^ = 18.8 *p* = 0.01 Significant						
Uptake	CPI 0	CPI 1	CPI 2	CPI 3	CPI 4	
Yes	6 (46.2)	113 (72.0)	160 (84.2)	73 (93.6)	12 (75.0)	364
No	7 (53.8)	44 (28.0)	30 (15.8)	5 (6.4)	4 (25.0)	90
Total	13 (100)	157 (100)	190 (100)	78 (100)	16 (100)	454
X^2^ = 24.4 *p* = 0.00 Significant						

CPI = Community Periodontal Index

CPI Score 0 = Healthy periodontium; CPI Scores 1 & 2 = Gingivitis; CPI Score 3 & 4 = Periodontitis

**Table 4:** Percentage difference in demand and uptake of dental scaling

Groups	% requesting or taking up scaling		Outcome
	Demand	Uptake	
For all participants	75/454 = 16.5%	364/454 = 80.2%	80.2–16.5 = 63.7% difference
For those with Scaling treatment need^a^	9/94 = 9.6%	85/94 = 90.4%	90.4–9.6 = 80.8% difference

^a^This group was made up of participants with CPI Scores of 3 and 4

Using the number of dental visits prior to outreach as a proxy for awareness in our study, a multiple regression analysis of factors affecting oral health awareness was done. Analysis showed that there was 0.279 increase in awareness depending on the place of abode of the respondents (*p* = 0.00), so we can predict that the geographic location (urban or rural) of a person affects the oral health awareness. Our table also showed that for every unit increase in age there was a 0.004 reduction in number of dental visits (*p* = 0.853) and a 0.024 increase in uptake of S&P is predicted (*p* = 0.652) (Table 5).

**Table 5:** A multiple regression of factors affecting oral health awareness

Variables	Standardized coefficient	*P* value	Lower limit (Confidence Interval)	Upper limit (Confidence Interval)
Constant	.520	0.00	.252	.789
Uptake of S&P	0.024	.652	−.082	.131
No. of times brushed	0.047	.243	−.032	.125
Age category	−0.004	.853	−.049	.041
Demand for S&P	0.063	.293	−.055	.181
Gender	0.066	.140	−.022	.154
Location	.279	.0001	.183	.375

R^2^ = 0.116

Dependent variable: Number of visit to dentist (as measure of awareness)

Predictors (constant), uptake of S&P, no. of times brushed, age category, demand for S&P, Gender and Location

Predictors coded as yes = 1; No = 2

Reference categories: No uptake of S&P, No brushing, Age category: 0 to 39 yrs., No demand for S&P, Gender- Male, For location- Rural, Unit of variables have been standardized

## Discussion

The ultimate aim of periodontal treatment is to control disease progression or achieve a rate of progression which is compatible with a functional dentition for the lifetime of the individual [2, 4]. Our study created an opportunity for such through oral health education as intervention and platform for interaction between populace and the dental professionals in the communities. It also served as an opportunity to introduce basic dental scaling to the people who though needed the service were yet to attend dental clinic.

From our study we observed that more females than males enlisted in the study. However, this higher attendance of women than men does not necessarily translate to increased awareness because the effect of gender on awareness was found not to be significant in this study. This could mean that females were probably more readily available at the time of our visit or that given the fact that women are the primary care givers in the home, and tend to visit health facilities more than males, either for themselves or their children; they had better opportunity to partake than the males**.** A previous study on gender influence on oral health proposed that females are more informed about oral health than men and take more interest in their oral health than men [29]. In as much as we do not disagree with this notion, most public or community dental practices are usually incorporated in a regular medical facility and as such women who visit for other purposes such as maternal and child health issues, are readily available for dental awareness creation programs. Therefore, they were in a better position to get more information about preventive oral health services.

We observed that only a minority of the participants with scaling treatment need (9.6%) demanded for scaling services at the outreaches, however post-intervention, we recorded an uptake of scaling by 90.4% of participants which most likely was spurred up by some factors (see Table 4). We attributed this mainly to the motivation and educational talks received during the program having positive effect on participants. This corroborates the statement by Nash and Brown (2012) that “Oral disease and the resulting need for information, therapy, and rehabilitation are the starting point for the demand for dental services” [30]. Our oral health intervention interlaced with the supply of the scaling services to the participants might have addressed the barriers posed by availability, and access to treatment similar to reports from other studies [31, 32]. Access can no longer be looked at from the patients’ ability to obtain or utilize care alone but is now essentially a concept of supply - demand where both availability of dental care which can represent the supply side and individual factors related to patients need, cultural and community considerations relating to the demand side are both taken into consideration [33]. On the overall, with a recorded percentage difference of 80.8% in demand and uptake of scaling for those with scaling treatment need and 63.7% all participants (Table 4**),** we can infer that the intervention in this study positively influenced uptake of scaling. This is similar to the report of a study in school aged children that OHE is effective in increasing knowledge, attitude and practice of individuals [34].

Looking at gender influence on demand and uptake of scaling, males demanded for scaling of teeth more than women but more women took up the scaling treatment at the long run. This may be due to cultural and behavioural attributes of men where they are initially more decisive than women. However, the actual uptake of healthcare is subject to a myriad of factors such as their workplace demands, self- perceived oral health need, and their perception of the seriousness of the condition [32, 35, 36]. This is corroborated by a report that females had better oral healthcare habits than the males, were more concerned about how their teeth looked than males, thus would be more inclined to get their teeth scaled and polished and retain their teeth in good health [37].

Furthermore, we observed that individuals with CPI scores 3 and 4, that is, more severe periodontal conditions, were not interested in S & P; we attributed this to lack of perceived need for it. In our environment, credible reports show perceived need of dental condition is a function of how aware the individuals are about oral health or health in general. [32, 35]. Awareness creation can motivate behavioural change in respondents and improve their dental health seeking pattern as reported in other studies. [14] It is our view that the oral health education created a platform to motivate those with severe periodontal condition who initially did not demand for scaling to take up.

Furthermore, a number of factors affected oral health awareness in the present study (Table 5). The location of the participants, that is rural or urban, affected awareness significantly. This observation also flows with our other finding stated above that more urban participants than rural demanded for scaling (*p* = 0.00). Rural dwellers have been known to face challenges of awareness and use of oral health facilities [14, 22]. By implication, low oral health awareness has a direct effect on the illness seeking behaviour of the individuals and population and need to be built up in order to motivate the use of dental services; our study was able to achieve this to a reasonable extent. Other factors such as age, gender and number of times brushed, illustrated a trend in influencing the outcome variable, but the results were not statistically significant.

The present study has limitations that must be taken into account to correctly interpret the findings. First, the use of prior dental visit alone as proxy for awareness may lead to partial assessment of oral health awareness as other facets exist but within the scope of our study, we were able to synergize the two. Secondly, using community outreach programs to recruit study participants could be a limitation for our study. However, this method has been known to aid recruitment of hard to reach populations or minority groups into studies. In the light of this, our approach was able to capture women, children as well as men who most often do not seek healthcare [38]. This approach has proved effective in other studies as a means of recruiting study participants. [21, 22]. Another limitation to our study could be the use of CPI to measure periodontal status of participants. CPI is saddled with the challenge of either underestimating or overestimating periodontal treatment needs as fake pockets resulting from gingival overgrowth without attachment loss could be mistaken for true periodontitis.

In terms of strengths, we were able to reach out to a good number of people especially the grassroots, hoist promotional activities like oral health education, and provide professional dental scaling of teeth to the individuals within the ambit of our study. These gains could be sustained by instituting appropriate health policies which will inform better planning and encourage the viability of oral health care activities in the communities possibly by incorporating them into existing primary health care centres.

## Conclusions

The demand for scaling and polishing by the participants was sub-optimal; uptake during and after the outreaches was however satisfactory. The keenness to take up S&P suggests benefits accruing from the oral health education as intervention in our study. Only geographic location of the participants affected their oral health awareness significantly; other factors such as age and gender did not. Our findings could aid in appropriate policy making and planning for basic oral health services to reach the grassroots possibly using existing primary oral health care platforms.

## Additional files

Additional file 1: Map of Enugu State. Map of Nigeria highlighting Enugu State and Map of Enugu with 17 LGAS. (DOCX 52 kb)

Additional file 2: Questionnaire. Date collection tool. (DOCX 21 kb)

## Abbreviations

CPI: Community Periodontal Index
OHE: Oral health Education
S&P: Scaling and Polishing
WHO: World Health Organization
