# Supplementary material for: Using community participation to assess demand and uptake of scaling and polishing in rural and urban environments
Source: BMC Oral Health. 2018 May 10;18:80. doi: 10.1186/s12903-018-0548-9 (PMC5946404; doi:10.1186/s12903-018-0548-9)

Map of Nigeria highlighting Enugu State in red


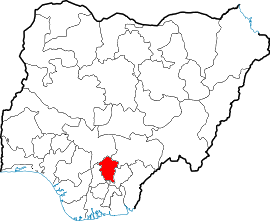


Map of Enugu State showing Local Government Areas


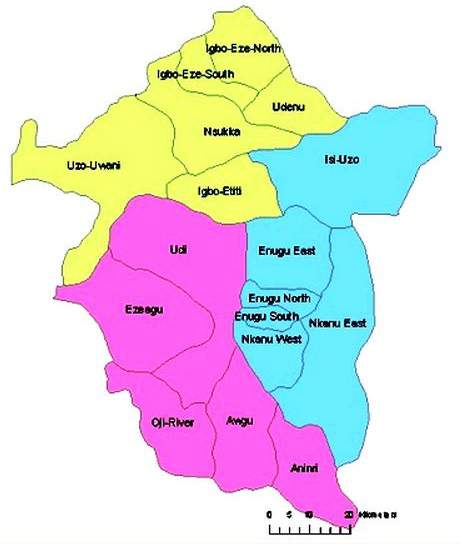

Supplement: Supplementary file 1 — Map of Enugu State. Map of Nigeria highlighting Enugu State and Map of Enugu with 17 LGAS. (DOCX 52 kb) [file 12903_2018_548_MOESM1_ESM.docx]
