# Supplementary material for: Using community participation to assess demand and uptake of scaling and polishing in rural and urban environments
Source: BMC Oral Health. 2018 May 10;18:80. doi: 10.1186/s12903-018-0548-9 (PMC5946404; doi:10.1186/s12903-018-0548-9)
Supplement: Supplementary file 2 — Questionnaire. Date collection tool. (DOCX 21 kb) [file 12903_2018_548_MOESM2_ESM.docx]

**DEPARTMENT OF PREVENTIVE DENTISTRY**

**UNIVERSITY OF NIGERIA**

The members of staff of the above named Department are conducting a study. The information you are going to provide is for research purposes only and will be treated confidentially. You do not need to mention your name and address to ensure confidentiality. Please answer honestly.

Please note that you may **consent** to fill the questionnaire to participate or refuse to fill if you do not want to participate. Thank you.

**SECTION A: DEMOGRAPHIC DATA**

1. How old are you? _______________
2. Sex: Male [ ] Female [ ]
3. Location: Urban area [ ] Rural [ ]

**SECTION B: DENTAL HISTORY**

1. Have you been to Dentist before this outreach? Yes [ ] No [ ]
2. If yes, how many times? Once [ ] Twice [ ] > Twice [ ]
3. What dental complaint do you have now?

Gum Bleeding [ ] Toothache [ ] Swelling [ ] Mobility [ ] Check up [ ]

Others (Please specify) _______________________

1. What dental concern/need do you want us to address today:

a. To clean my teeth [ ]

b. To remove my tooth/teeth [ ]

c. To fill my tooth/ teeth [ ]

d. To replace my tooth/teeth [ ]

e. Others (pls specify)

1. Have you had any dental treatment before? Yes [ ] No [ ]

If yes, please specify ____________________

1. What do you use to clean your teeth? ____________________
2. How many times a day do you clean teeth? ___________________

**SECTION C: DENTAL / PERIODONTAL EXAMINATION** (Please do not fill here, for researchers use only)

1. Teeth present:

|  |  |
| --- | --- |
|  |  |

1. Carious teeth

|  |  |
| --- | --- |
|  |  |

1. Missing teeth

|  |  |
| --- | --- |
|  |  |

1. Filled teeth

|  |  |
| --- | --- |
|  |  |

1. DMFT Score _________________
2. CPI Charting

|  |  |  |
| --- | --- | --- |
|  |  |  |

1. CPI Score ____________________

19. Treatment plan: Need S&P [ ] Does not need S&P [ ]

20. Treatment done: S&P [ ] No S&P [ ]
